# A pancancer analysis of the oncogenic role of cyclin B1 (CCNB1) in human tumors

**DOI:** 10.1038/s41598-023-42801-y

**Published:** 2023-09-27

**Authors:** Peng Dai, Lecai Xiong, Yanhong Wei, Xiaoyan Wei, Xuefeng Zhou, Jinping Zhao, Hexiao Tang

**Affiliations:** 1 Department of Thoracic Surgery, Zhongnan Hospital of Wuhan University, Wuhan, 430071 China; 2 Department of Cardiovascular Surgery, Zhongnan Hospital of Wuhan University, Wuhan, 430071 China; 3 Department of Rheumatology and Immunology, Zhongnan Hospital of Wuhan University, Wuhan, 430071 China; 4 Liyuan Cardiovascular Center, Liyuan Hospital, Tongji Medical College, Huazhong University of Science and Technology, Wuhan, 430077 China

**Keywords:** Lung cancer, Tumour biomarkers

## Abstract

Aberrant levels of the G2/M cyclin cyclin B1 (gene CCNB1) have been associated with multiple cancers; however, the literature lacks a focused and comprehensive analysis of the regulation of this important regulator of cell proliferation in cancer. Through this work, we performed a pancancer analysis of the levels of CCNB1 and dissected aspects of regulation and how this correlates with cancer prognosis. We comprehensively evaluated the expression and promoter methylation of CCNB1 across 38 cancers based on RNA sequencing data obtained from the Cancer Genome Atlas (TCGA). The correlation of CCNB1 with prognosis and the tumor microenvironment was explored. Using lung adenocarcinoma data, we studied the potential upstream noncoding RNAs involved in the regulation of CCNB1 and validated the protein levels and prognostic value of CCNB1 for this disease site. CCNB1 was highly expressed, and promoter methylation was reduced in most cancers. Gene expression of CCNB1 correlated positively with poor prognosis of tumor patients, and these results were confirmed at the protein level using lung adenocarcinoma. CCNB1 expression was associated with the infiltration of T helper cells, and this further correlated with poor prognosis for certain cancers, including renal clear cell carcinoma and lung adenocarcinoma. Subsequently, we identified a specific upstream noncoding RNA contributing to CCNB1 overexpression in lung adenocarcinoma through correlation analysis, expression analysis and survival analysis. This study provides a comprehensive analysis of the expression and methylation status of CCNB1 across several forms of cancer and provides further insight into the mechanistic pathways regulating Cyclin B1 in the tumorigenesis process.

## Introduction

The mechanism of tumorigenesis is very complicated, and genetic changes play an important role in it. In recent years, whole-genome sequencing analysis has furthered the understanding of tumor molecular mechanisms and has played an important role in promoting genome-driven oncology care^1,2^. There may be some common mechanisms for the occurrence and development of different tumors, and it is meaningful to perform a pancancer expression analysis of different genes and explore their correlation with clinical characteristics and potential molecular mechanisms. The Cancer Genome Atlas (TCGA) database contains DNA, RNA, protein, and epigenetic datasets, thus allowing us to perform pancancer analysis^3^.

The cell cycle-related protein cyclin B1 (CCNB1) is an important member of the cyclin family, which refers to proteins that differ in their levels to activate specific cyclin-dependent kinases (CDKs) required for progression in the cell cycle^4^. Activated CCNB1 can promote cells to enter the M phase from the G2 phase and initiate mitotic progression^5^. An increasing number of studies have shown that CCNB1 is closely related to the abnormal proliferation of cells and the occurrence of tumors, such as CCNB1 overexpression in liver cancer, breast cancer, esophageal cancer, and cervical cancer^6,7^. However, there is still a lack of pancancer analysis on the relationship between CCNB1 and various tumors based on clinical databases. In this study, we first constructed a pancancer analysis of CCNB1 based on the TCGA database. Gene expression, tumor stage, survival status, immune infiltration and other factors were included in the analysis to explore the potential molecular mechanism of CCNB1 in different cancers.

## Materials and methods

### Gene expression analysis

The TIMER2 website (http://timer.cistrome.org/)^8^ was used to analyze the expression difference of CCNB1 in different tumors and adjacent normal tissues of the TCGA database. For some tumors without normal adjacent tissues in TIMER2, such as adrenocortical carcinoma (ACC), lymphoid neoplasm diffuse large B-cell lymphoma (DLBC), and testicular germ cell tumors (TGCT), the GEPIA2 (http://gepia2.cancer-pku.cn/#analysis)^9^ website was used to compare the expression of CCNB1 in tumor tissues and adjacent normal tissues and obtain box plots. In addition, violin plots of CCNB1 expression in different pathological stages of all TCGA tumors were obtained in the “Stage Plot” module of GEPIA2.

### Survival prognosis analysis

The “survival analysis” module of GEPIA2 was used to obtain the overall survival (OS) and disease-free survival (DFS) significance map data of CCNB1 in all TCGA tumors, with 50% as the cutoff to split the high-expression and low-expression cohorts.

### DNA methylation and immune infiltration analysis

The UALCAN portal (http://ualcan.path.uab.edu/analysis.html) was used to explore the DNA methylation level of CCNB1 between TCGA tumors and corresponding normal tissues. The “Immune-Gene” module of TIMER2 was used to explore the association between CCNB1 expression and immune infiltrates across all TCGA tumors. The P values and partial correlation values were obtained via the purity-adjusted Spearman’s rank correlation test.

### CCNB1-related gene enrichment analysis

The available experimentally determined CCNB1-binding proteins were obtained from the STRING website (https://string-db.org/) with the following main parameters: minimum required interaction score “Low confidence (0.150)”, meaning of network edges “evidence”, active interaction sources (“experiments”) and max number of interactors to show “no more than 50 interactors”.

The “Similar Gene Detection” module of GEPIA2 was used to obtain the top 100 CCNB1-correlated targeting genes based on the TCGA database. Intersecting genes between CCNB1-binding proteins and the top 100 CCNB1-related genes were obtained through a Venn diagram. Kyoto Encyclopedia of Genes and Genomes (KEGG)^10,11^ pathway (Permission ID: 231307) and Gene Ontology (GO) analyses of the intersecting genes were performed and visualized with a bubble diagram. Finally, we showed the partial correlation and P value of intersecting genes in the purity-adjusted Spearman’s rank correlation test through a heatmap obtained from TIMER2 and performed survival analysis on all intersecting genes in Kaplan‒Meier Plotter (http://kmplot.com/analysis/index.php?p=service&cancer=pancancer_rnaseq).

### Candidate microRNA prediction

The StarBase database (http://starbase.sysu.edu.cn/) was employed to predict the upstream binding miRNAs of CCNB1, and only the predicted microRNAs that commonly appeared in more than two programs (PITA, RNA22, miRmap, microT, miRanda, PicTar, and TargetScan) were included for subsequent analyses. Subsequently, the prognostic values of selected microRNAs were analyzed by the Kaplan‒Meier Plotter database, and candidate microRNAs were finally determined.

### Identification of upstream long noncoding RNAs (lncRNAs) of candidate microRNAs

The predicted upstream lncRNAs of candidate microRNAs were obtained from the StarBase database. According to the competing endogenous RNA (ceRNA) hypothesis, lncRNAs were selected based on a negative relationship with candidate microRNAs (P value < 0.05, correlation coefficient <  − 0.1) and a positive relationship with CCNB1 (P value < 0.05, correlation coefficient > 0.1) and were chosen based on the prognostic values determined by Kaplan‒Meier Plotter.

### Patient tissue specimens

Tissue specimens from 60 LUAD patients and 11 benign pulmonary lesion (BPL) patients who underwent surgery from April 2014 to July 2017 were collected for tissue chips. Written informed consent was obtained from each patient (Supplementary File). None of the patients had a history of preoperative chemotherapy or radiotherapy. The project was approved by the Ethics Committee of Zhongnan Hospital of Wuhan University (approval number: 2021100K), and the informed consent requirement was waived. All methods were performed in accordance with the relevant guidelines and regulations.

### Statistical analysis

Most of the statistical analyses in this study were calculated by the online database or tools mentioned above. A P value < 0.05 or log rank P value < 0.05 was considered statistically significant.

## Results

### Pancancer analysis of CCNB1 expression

**Figure 1:**
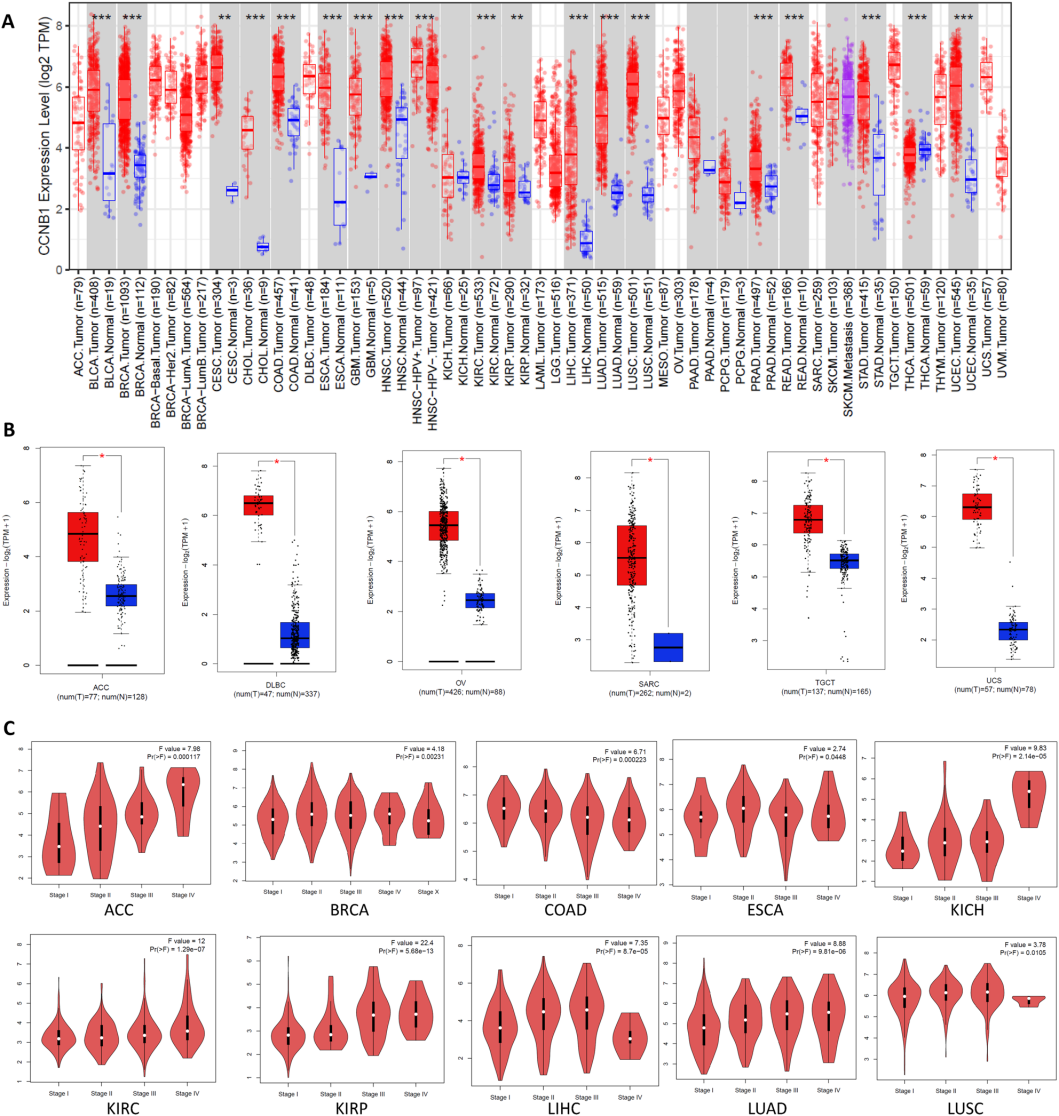
Expression level of the CCNB11 gene in different cancers and pathological stages. (**A**) The expression levels of CCNB1 in different cancers or specific cancer subtypes were analyzed through TIMER2. *P < 0.05; **P < 0.01; ***P < 0.001. Red indicates tumor tissue, and blue indicates normal tissue. (**B**) The expression of CCNB1 in certain cancers without corresponding normal tissues in the TCGA project. *P < 0.05; **P < 0.01; ***P < 0.001. Red indicates tumor tissue, and blue indicates normal tissue. (**C**) Based on the TCGA data, the expression levels of CCNB1 were analyzed by the main pathological stages of ACC, BRCA, COAD, ESCA, KICH, KIRC, KIRP, LIHC, LUAD, and LUSC. Log2 (TPM + 1) was applied for log-scale. The ordinate is the expression amount of CCNB1.

The expression differences of CCNB1 between tumor and adjacent normal tissues for the different tumors of the TCGA database are shown in Fig. 1A. For certain tumors without corresponding normal tissues in TCGA, the expression differences in the GTEx (Genotype-tissue expression) database were analyzed, and box plots were obtained via the GEPIA2 website (Fig. 1B). According to the results, CCNB1 was significantly upregulated in 23 cancer types compared with corresponding normal tissues, including adrenocortical carcinoma (ACC), bladder urothelial carcinoma (BLCA), bladder urothelial carcinoma (BRCA), cervical squamous cell carcinoma (CESC), cholangiocarcinoma (CHOL), colon adenocarcinoma (COAD), lymphoid neoplasm diffuse large B-cell lymphoma (DLBC), esophageal carcinoma (ESCA), glioblastoma multiforme (GBM), head and neck squamous cell carcinoma (HNSC), kidney renal clear cell carcinoma (KIRC), kidney renal papillary cell carcinoma (KIRP), liver hepatocellular carcinoma (LIHC), lung adenocarcinoma (LUAD), lung squamous cell carcinoma (LUSC), ovarian serous cystadenocarcinoma (OV), prostate adenocarcinoma (PRAD), rectum adenocarcinoma (READ), sarcoma (SARC), stomach adenocarcinoma (STAD), testicular germ cell tumors (TGCT), uterine corpus endometrial carcinoma (UCEC), and uterine carcinoma (UCS).

Additionally, violin plots of CCNB1 expression in different pathological stages of all TCGA tumors were obtained via the “Pathological Stage Plot” module of HEPIA2. As shown in Fig. 1C, CCNB1 was significantly related to pathological stages in 10 types of tumors, including ACC, BRCA, COAD, ESCA, KICH, KIRC, KIRP, LIHC, LUAD, and LUSC.

### The prognostic values of CCNB1 in pancancer

**Figure 2:**
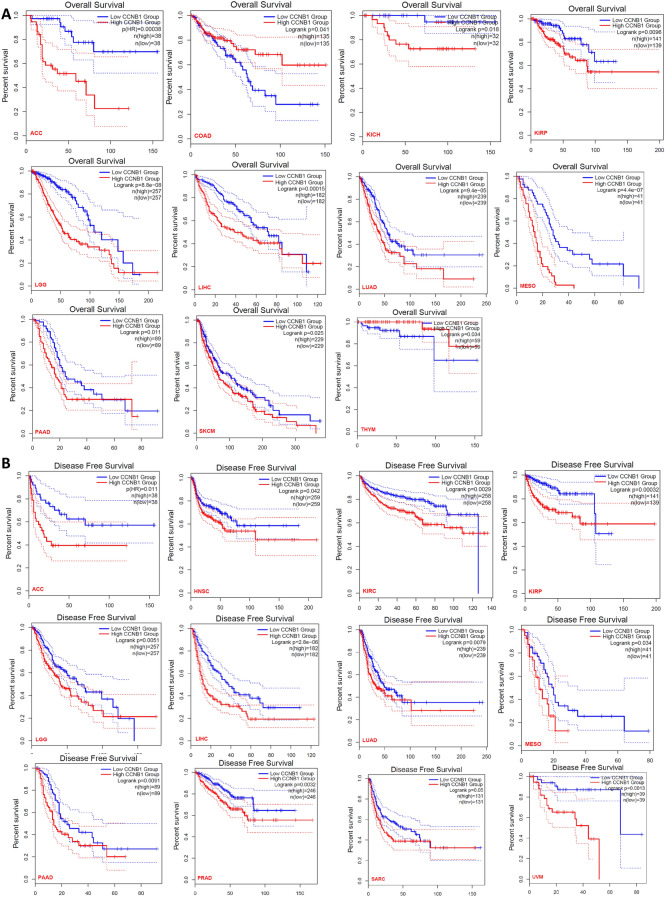
The prognostic value of CCNB1 for cancers in TCGA. The GEPIA2 tool was used to perform overall survival (**A**) and disease-free survival (**B**) analyses of different cancers in TCGA by CCNB1 expression. The Kaplan‒Meier curves with positive results are given.

Survival analysis for CCNB1 in pancancer was conducted. Overall survival and disease-free survival were included. As shown in Fig. 2, highly expressed CCNB1 was linked to poor prognosis of OS for tumors of ACC (P < 0.001), KICH (P = 0.0018), KIRP (P = 0.0096), LGG (P < 0.001), LIHC (P < 0.001), LUAD (P < 0.001), MESO (P < 0.001), PAAD (P = 0.011), and SKCM (P = 0.025) within the TCGA project, while COAD and THYM patients with higher expression of CCNB1 indicated better prognosis (P = 0.041, 0.034). The DFS analysis data showed that high CCNB1 expression indicated poor prognosis among all cancer types (ACC (P = 0.011), HNSC (P = 0.042), KIRC (P = 0.0029), KIRP (P < 0.001), LGG (P = 0.0051), LIHC (P < 0.001), LUAD (P = 0.0079), MESO (P = 0.034), PAAD (P = 0.0091), PRAD (P = 0.0032), SARC (P = 0.05), and UVM (P = 0.0013)).

### Enrichment analysis of CCNB1-related genes

**Figure 3:**
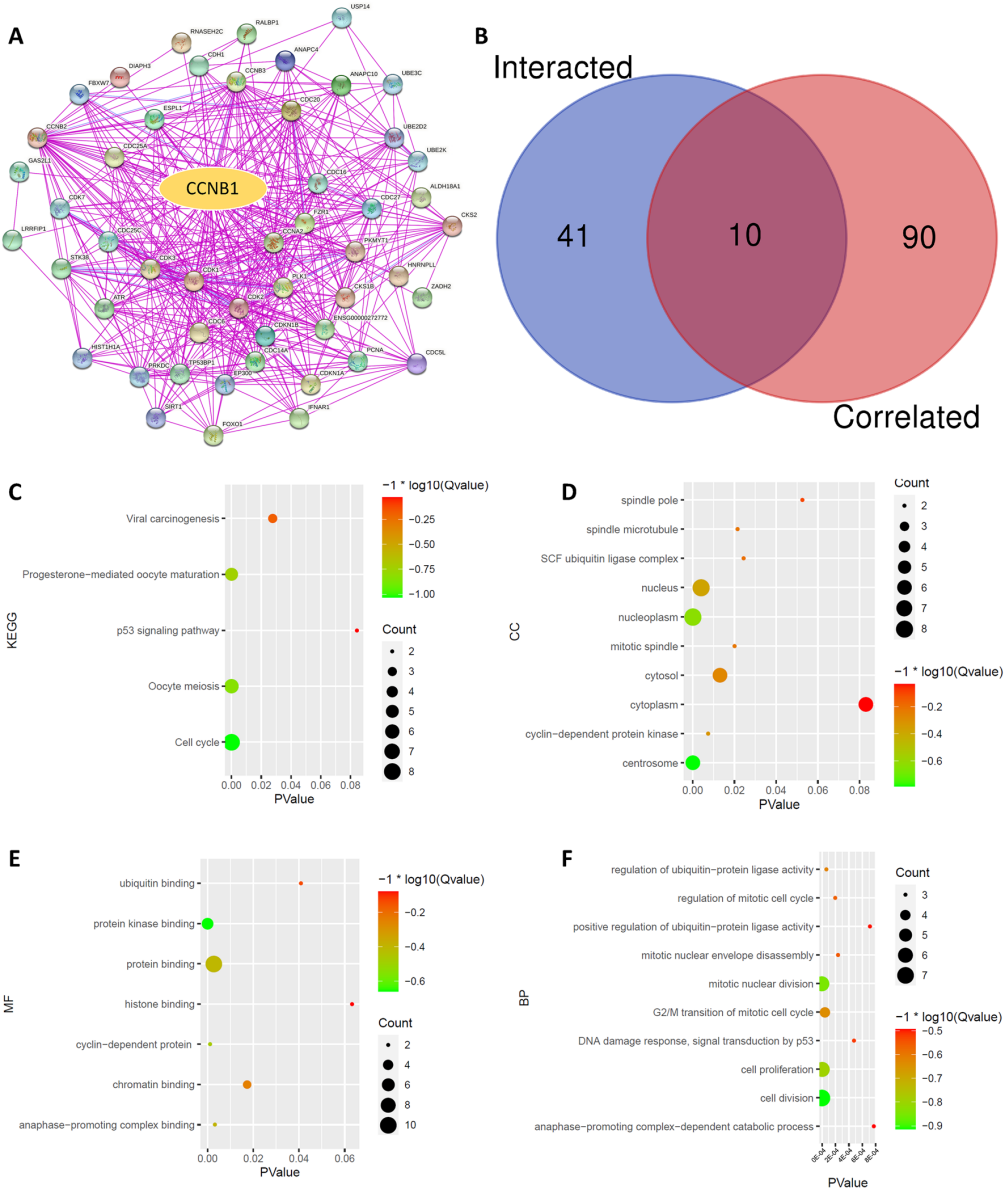
CCNB1-related gene enrichment analysis. (**A**) Fifty-one experimentally determined CCNB1-binding proteins were identified via the STRING tool. (**B**) An intersection analysis of CCNB1-binding and the top 100 CCNB1-correlated genes was conducted, and 10 intersecting genes were obtained. (**C**) Based on the 10 intersecting genes, KEGG pathway analysis was performed. (**D–F**) The top 10 items of GO analysis: biological processes (**F**), cellular components (**D**), and molecular functions (**E**) of the 10 intersecting genes.

To further explore the molecular mechanism of CCNB1 in tumorigenesis, we attempted to identify CCNB1-binding proteins and SND1^12^ expression-correlated genes for KEGG pathway and GO enrichment analyses. A total of 51 CCNB1-binding proteins supported by experimental evidence were obtained on the STRING website, and the protein‒protein interaction (PPI) network is shown in Fig. 3A. The top 100 CCNB1-related genes were obtained via the GEPIA2 tool. As shown in Fig. 3B, 10 intersecting genes (CCNA2, CCNB2, CDC20, CDC25C, PLK1, CDK1, CKS1B, ESPL1, CKS2, PCNA) between CCNB1-binding proteins and the top 100 CCNB1-related genes were obtained through a Venn diagram. The KEGG analysis of intersecting genes indicated that CCNB1 was mainly involved in the “cell cycle” in tumor pathogenesis (Fig. 3C). The results of GO analysis further indicated that most of the intersecting genes were involved in cell proliferation, cell cycle, cell division, and others (Fig. 3D–F). As shown in Fig. S3A, the expression level of CCNB1 was positively correlated with all 10 intersecting genes. Further survival analysis showed that high expression of all intersection genes was associated with poor prognosis of lung adenocarcinoma (Fig. S3B,C), and similar results were observed in KIRP and LIHC (Fig. S4).

### Prediction and analysis of upstream miRNAs of CCNB1

According to the StarBase database, 11 upstream microRNAs were identified.

**Figure 4:**
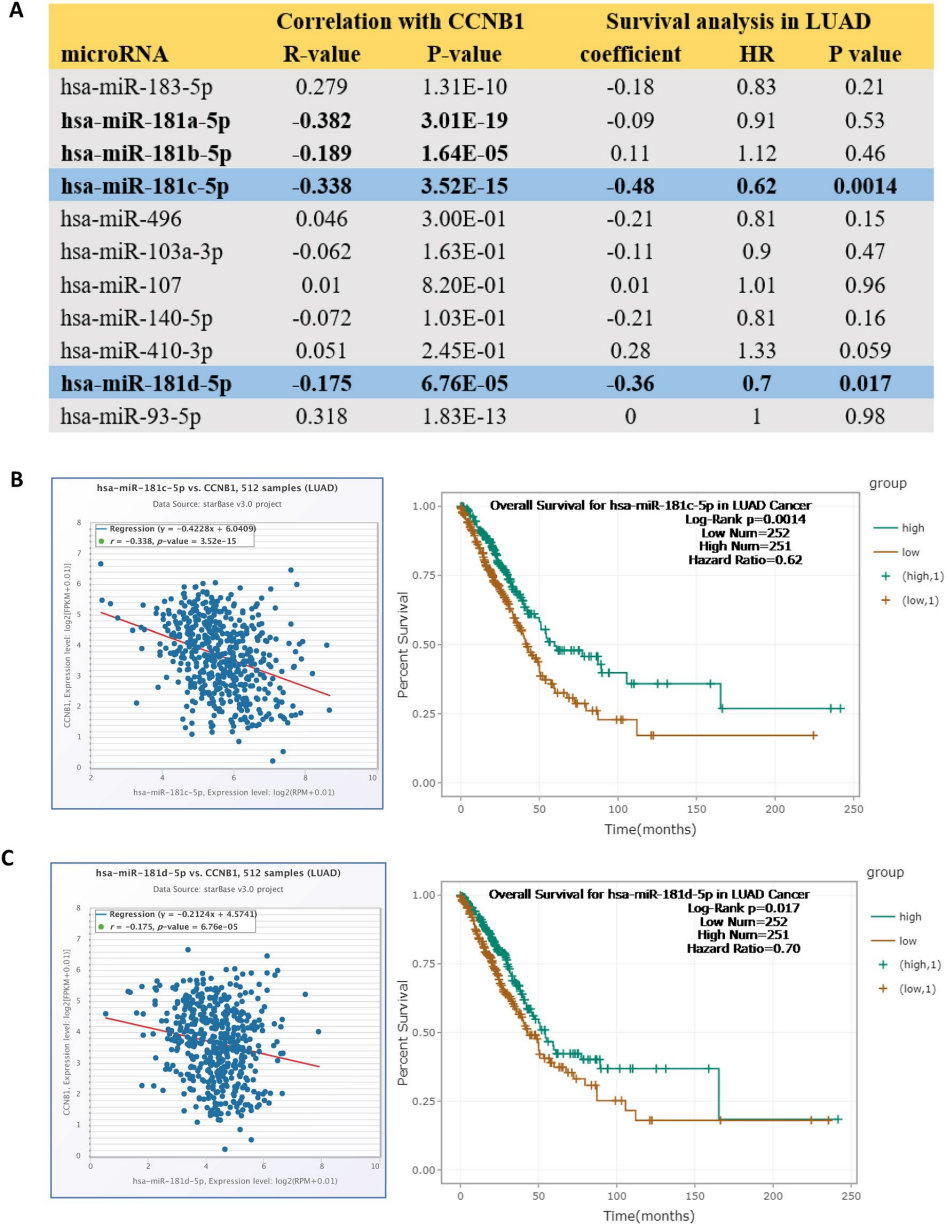
Identification of hsa-miR-181c-5p and hsa-miR-181d-5p as potential upstream microRNAs of CCNB1 in LUAD. (**A**) The expression correlation between predicted microRNAs and CCNB1 in LUAD analyzed by the starBase database. (**B,C**) The expression of hsa-miR-181c-5p and hsa-miR-181d-5p in lung adenocarcinoma was negatively correlated with CCNB1 (all P < 0.001), and high expression was associated with a better prognosis (P = 0.0014, 0.017).

According to the mechanism by which miRNAs regulate target gene expression, upstream microRNAs should be negatively correlated with CCNB1. As shown in Fig. 4A, CCNB1 was significantly negatively correlated with hsa-miR-181a-5p, hsa-miR-181b-5p, hsa-miR-181c-5p and hsa-miR-181d-5p and positively correlated with hsa-miR-183-5p and hsa-miR-93-5p. To further determine the upstream microRNAs, the correlation between microRNAs and the prognosis of lung adenocarcinoma was explored. The results showed that low expression of hsa-miR-181c-5p and hsa-miR-181d-5p was associated with poor prognosis of lung adenocarcinoma (P = 0.0014, 0.017, Fig. 4A,C). The above results suggested that hsa-miR-181c-5p and hsa-miR-181d-5p might be the most potential upstream microRNAs of CCNB1 in LUAD.

### Prediction and analysis of upstream lncRNAs of hsa-miR-181c-5p and hsa-miR-181d-5p

**Figure 5:**
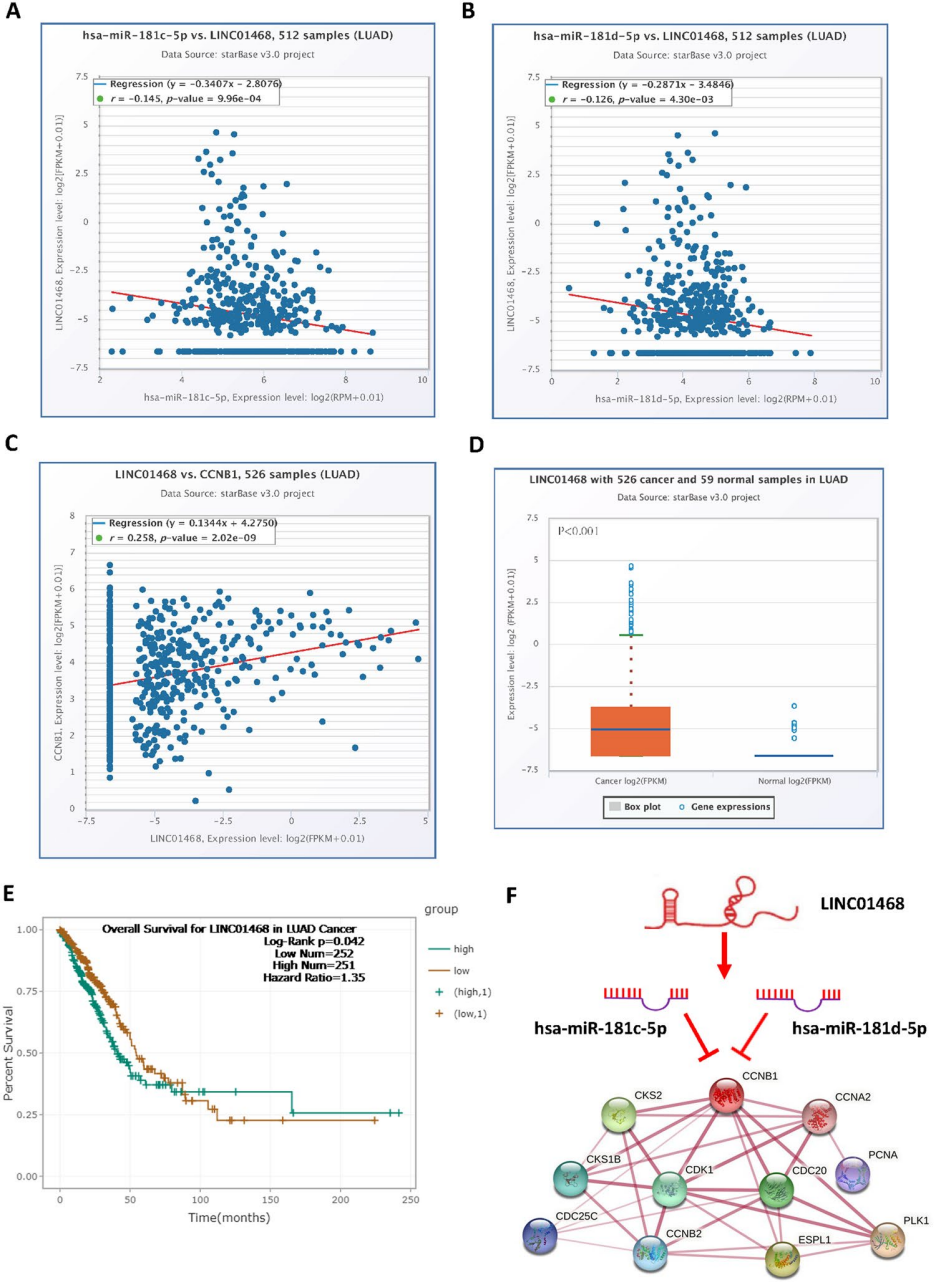
Identification of LINC01468 as the potential upstream lncRNA of hsa-miR-181c-5p and hsa-miR-181d-5p in LUAD. Correlation analysis showed that the expression of LINC01468 in LUAD was negatively correlated with hsa-miR-181c-5p (**A**) and hsa-miR-181d-5p (**B**) but positively correlated with CCNB1 (**C**). (**D**) The expression of LINC01468 in LUAD was significantly higher than that in control normal samples. (**E**) The results of overall survival analysis showed that high expression of LINC01468 was related to poor prognosis. (**F**) CCNB1-related ceRNA regulation network.

The upstream lncRNAs of hsa-miR-181c-5p and hsa-miR-181d-5p were predicted via the StarBase database. A total of 58 possible lncRNAs were forecasted. Based on the competing endogenous RNA (ceRNA) hypothesis, lncRNAs can increase the expression of target mRNAs by competitively binding to microRNAs. Therefore, LINC01468, which was not only negatively related to microRNA but also positively related to CCNB1, was chosen. As shown in Fig. 5B, LINC01468 was negatively related to hsa-miR-181c-5p (R =  − 0.145, P < 0.001, Fig. 5A) and hsa-miR-181d-5p (R =  − 0.126, P = 0.0043, Fig. 5B) and positively related to CCNB1 (R = 0.258, P < 0.001, Fig. 5C). Similarly, LINC01468 was highly expressed in lung adenocarcinoma (P < 0.001, Fig. 5D), and high expression was associated with poor prognosis (P = 0.042, Fig. 5E). As shown in Fig. 5E, we constructed a protein network based on the ceRNA hypothesis. In this network, the overexpression of LINC01468 caused a decrease in hsa-miR-181c-5p and hsa-miR-181d-5p. The deletion of hsa-miR-181c-5p and hsa-miR-181d-5p promoted the translation of CCNB1, which promoted the progression of LUAD by regulating the cell cycle.

### DNA methylation and immune infiltration analysis

The DNA methylation level of CCNB1 between TCGA tumors and corresponding normal tissues was explored in the UALCAN portal. Contrary to the expression level of CCNB1, a reduced methylation level of CCNB1 was observed in most tumor tissues (BLCA, CESC, COAD, ESCA, HNSC, KIRC, KIRP, LIHC, LUAD, LUSC, PRAD, SARC, TGCT, THCA, UCEC) compared with corresponding normal tissues (Fig. S1). CCNB1 expression was positively related to Th2 cell infiltration in all tumors (Fig. S2A,B), and high Th2 cell infiltration in ACC, KIRC, LGG, LUAD, PAAD, and UVM suggested poor prognosis (Fig. S2B).

### Validation in LUAD tissue

**Figure 6:**
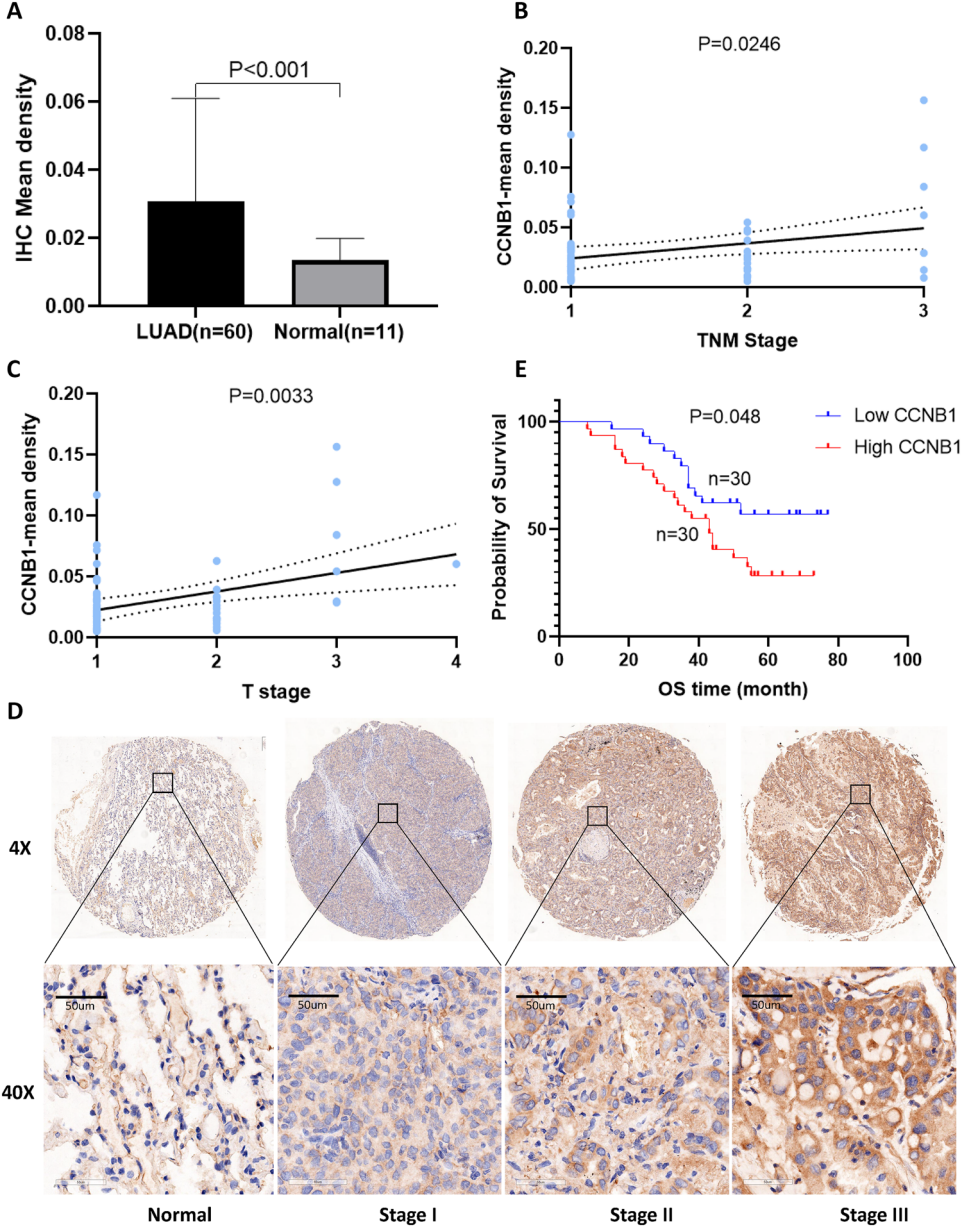
Validation of CCNB1 in LUAD. (**A**) Immunohistochemical results of the tissue chip showed that the expression level of CCNB1 in LUAD was significantly higher than that in normal tissues, and the expression of CCNB1 was correlated with TNM (**B**) and T stages (**C**). (**D**) Representative immunohistochemical staining showing CCNB1 expression in different LUAD stages. (**E**) The Kaplan‒Meier results showed that high CCNB1 expression was related to poor prognosis.

To verify the results of the above bioinformatics analysis, immunohistochemical (IHC) staining was performed on tissue microarray slides containing 60 LUAD tissues and 11 normal tissues. The results showed that CCNB1 was highly expressed in LUAD compared to normal tissue (P < 0.001, Fig. 6A,D). Furthermore, the expression of CCNB1 was related to TNM and T stage (P = 0.0246, 0.0033, Fig. 6B,C). Survival analysis in patients with LUAD showed that patients with high CCNB1 expression had a poorer prognosis (P = 0.048, Fig. 6E). The above results were all consistent with the results of the bioinformatics analysis.

## Discussion

Increasing evidence shows that CCNB1 is overexpressed in most human tumors^4^, which is consistent with the results of this study (Fig. 1A,B). Our study also showed that CCNB1 was significantly related to OS and DFS (Fig. 2). Recent studies have shown that in addition to directly regulating the cell cycle, CCNB1 can also coordinate the cell cycle by enhancing mitochondrial activity, which is closely related to the pathological characteristics of tumors^13^. A correlation between CCNB1 and tumor stage was observed in this study (Fig. 1C). We explored the potential mechanism of CCNB1 in tumors through the tumor microenvironment (TME) and DNA methylation.

TP53 gene mutations are observed in most human malignancies and play an important role in tumorigenesis^14^. Among the 10 CCNB1-related genes, CCNB2 and CDK1 participated in the P53 pathway according to the results of enrichment analysis (Fig. 3). A study in pancreatic cancer showed that CCNB1 silencing inhibited cell proliferation and induced cell apoptosis via the p53 signaling pathway^4,15^. A recent study indicated that P53 can inhibit the transcription of CCNB2, CDK, and other cyclin genes through the p53-p21-DREAM-CDE/CHR pathway, leading to cell cycle arrest and apoptosis^16^. Cyclin-dependent kinase 1 encoded by CDK1 is the only kinase essential for cell cycle regulation^17^. Cyclin A2 (CCNA2) is also an important molecule in the cyclin family. Studies in BRCA showed that the dysregulated expression of CDK1/CCNA2/CCNB1 was related to prognosis^18^. Other CCNB1-related genes have shown different diagnostic significance in different studies^19–23^.

Recent studies have fully confirmed the regulatory role of noncoding RNAs (microRNAs, lncRNAs, circular RNAs, etc.) in cancers^24^. Competing endogenous RNAs link the function of protein-coding mRNAs with noncoding RNAs and can regulate the expression level of each other by competing for shared microRNAs^25^. Based on the good performance of CCNB1 in multiple analyses of lung adenocarcinoma, we mainly explored the ceRNA regulatory mechanism of CCNB1 in lung adenocarcinoma. Hsa-miR-181c-5p and hsa-miR-181d-5p were recognized as CCNB1 upstream regulatory microRNAs in lung adenocarcinoma. Research has shown that hsa-miR-181c-5p is involved in the target pathway of cell proliferation and the cell cycle in pediatric cancer stem cells^26^. In breast cancer, head and neck squamous cell carcinoma, and other tumor studies, it has been shown that hsa-miR-181c-5p affects the prognosis of tumors by regulating the expression of multiple genes^27–29^. Research has shown that hsa-miR-181d-5p can repress the activation/phosphorylation of the transcription factors STAT3/5A and further inhibit the progression of glioblastoma^30^. Another in vitro study showed that overexpression of hsa-miR-181d significantly inhibits cell proliferation, migration, and cell cycle progression in esophageal squamous cell carcinoma^31^. Hsa-miR-181c-5p and hsa-miR-181d-5p both showed strong predictive effects on the prognosis of lung adenocarcinoma in this study (Fig. 4B,C). This study shows that LINC01468 is significantly related to the prognosis of lung adenocarcinoma. Similar results were observed in bladder urothelial carcinoma, and the research showed that the combined detection of LINC01468 and other lncRNAs had important predictive significance for bladder urothelial carcinoma^32^. According to the results of this study, hsa-miR-181c-5p and hsa-miR-181d-5p inhibited the expression of CCNB1 in LUAD, while LINC01468 promoted the expression of CCNB1 through competitive sharing of hsa-miR-181c-5p and hsa-miR-181d-5p.

In addition to genetics, epigenetic changes also play an important role in carcinogenesis, and DNA methylation is an important part of epigenetics^33^. DNA methylation is often described as a ‘silencing’ epigenetic mark and is involved in adding stability to the repression of transcription^34^. Changes in promoter methylation and histone acetylation levels are the result of imbalances in enzyme balance and ultimately lead to tumor progression^35^. According to the results of the study, CCNB1 was highly expressed in tumor tissues and was associated with a poor prognosis; therefore, we hypothesized that CCNB1 might be an oncogene. Compared with corresponding normal tissues, the promoter methylation level of CCNB1 in tumor tissues was significantly reduced (Fig. S1), which led to a decrease in the stability of the repression of transcription and ultimately led to an increase in the expression level of CCNB1 and promoted tumor progression.

The important role of the immune system in cancer control is beyond doubt^36^. Helper T cells (Th) are the key immune cells for regulating immunity and are divided into at least four subsets, T regulatory, Th1, Th2, and Th17 cells, which are jointly involved in regulating the immune response^37^. The results of this study showed that CCNB1 was positively correlated with Th2 cell infiltration in all TCGA tumors (Fig. S2A). Studies have shown that Th2 cells have both antitumor and protumor effects in the TME^38^. Th2 cell-mediated type 2 immune responses can contribute to antitumor immunity, while Th2 cells promote tumor progression through secreted cytokines^38–40^. In this study, high Th2 cell infiltration in ACC, KIRC, LGG, LUAD, PAAD, and UVM suggested a poor prognosis (Fig. S2B). The above results indicated that CCNB1 was related to Th2 cell infiltration in certain tumors, such as AAC and LUAD, and affected prognosis. In conclusion, our study indicated that the expression of CCNB1 is widely increased in many tumor types, and the expression is positively correlated with the poor prognosis of patients. At the same time, CCNB1 may be an oncogene, and it may affect the tumor microenvironment through Th2 cells. Furthermore, the LINC01468-hsa-miR-181c-5p/hsa-miR-181d-5p-CCNB1 network may play an important role in LUAD progression.

### Supplementary Information

Supplementary Figures.

## Data Availability

GEPIA2 (http://gepia2.cancer-pku.cn/#analysis), UALCAN portal (http://ualcan.path.uab.edu/analysis.html), STRING website (https://string-db.org/), Kaplan–Meier Plotter (http://kmplot.com/analysis/index.php?p=service&cancer=pancancer_rnaseq), StarBase database (http://starbase.sysu.edu.cn/).

## Abbreviations

ACC: Adrenocortical carcinoma
BPL: Benign pulmonary lesions
BLCA: Bladder urothelial carcinoma
BRCA: Bladder urothelial carcinoma
CCNB1: Cell cycle-related proteins cyclin B1
CDKs: Cyclin-dependent kinases
CeRNA: Competing endogenous RNA
CESC: Cervical squamous cell carcinoma
CHOL: Cholangio carcinoma
COAD: Colon adenocarcinoma
DFS: Disease-free survival
DLBC: Lymphoid neoplasm diffuse large B-cell lymphoma
ESCA: Esophageal carcinoma
GBM: Glioblastoma multiforme
GO: Gene Ontology
HNSC: Head and neck squamous cell carcinoma
KEGG: Kyoto encyclopedia of genes and genomes
KIRC: Kidney renal clear cell carcinoma
KIRP: Kidney renal papillary cell carcinoma
LIHC: Liver hepatocellular carcinoma
LncRNAs: Long noncoding RNAs
LUAD: Lung adenocarcinoma
LUSC: Lung squamous cell carcinoma
OV: Ovarian serous cystadenocarcinoma
OS: Overall survival
PRAD: Prostate adenocarcinoma
READ: Rectum adenocarcinoma
SARC: Sarcoma
STAD: Stomach adenocarcinoma
TCGA: The Cancer Genome Atlas
TGCT: Testicular germ cell tumors
UCEC: Uterine corpus endometrial carcinoma
UCS: Uterine carcinosarcoma
PPI: Protein‒protein interaction
IHC: Immunohistochemical
THYM: Thymoma
MESO: Mesothelioma
