# Supplementary figures and images for: A pancancer analysis of the oncogenic role of cyclin B1 (CCNB1) in human tumors
Source: Sci Rep. 2023 Sep 27;13:16226. doi: 10.1038/s41598-023-42801-y (PMC10533567; doi:10.1038/s41598-023-42801-y)

## Slide 1
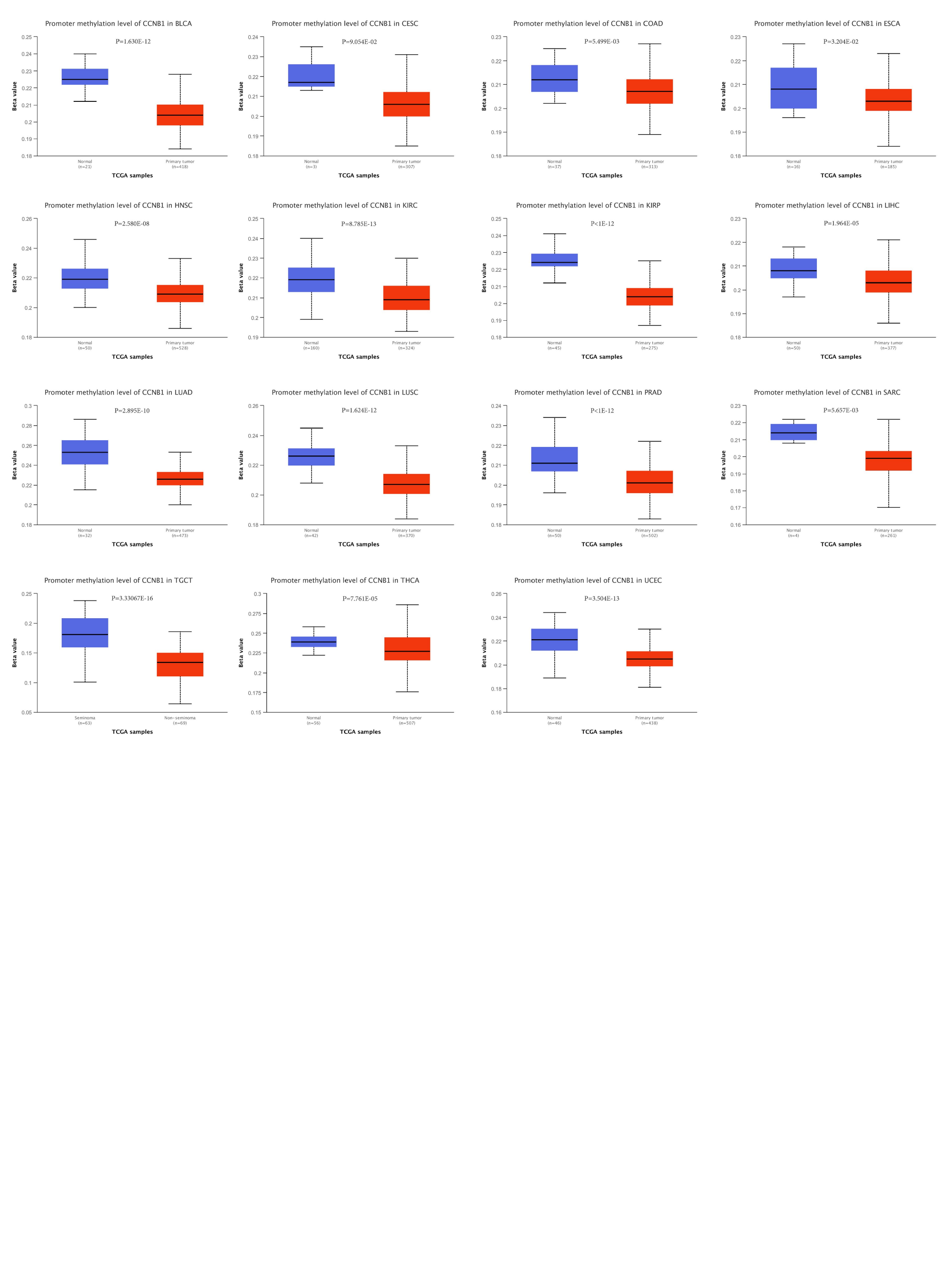

## Slide 2
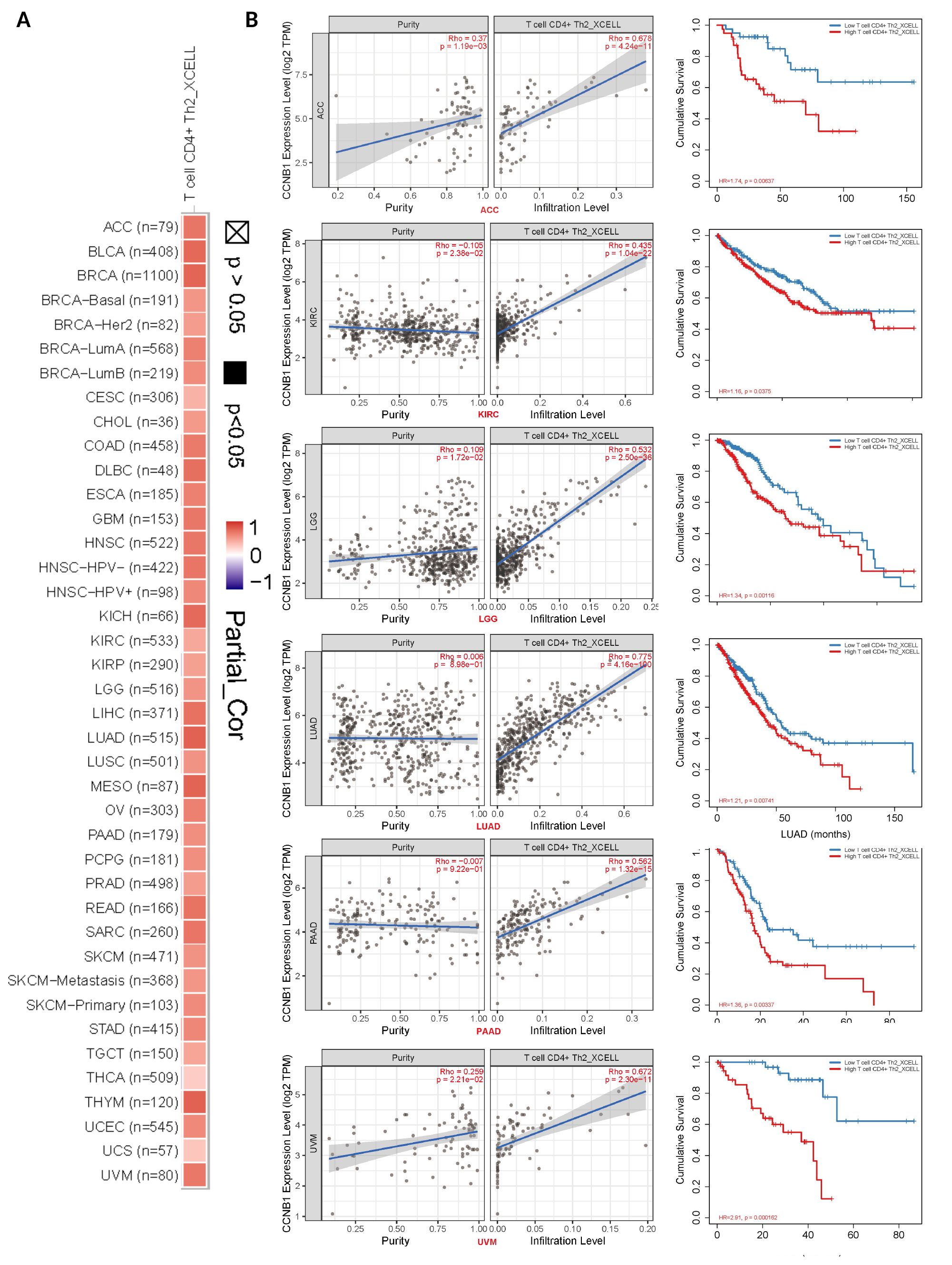

A
B

## Slide 3
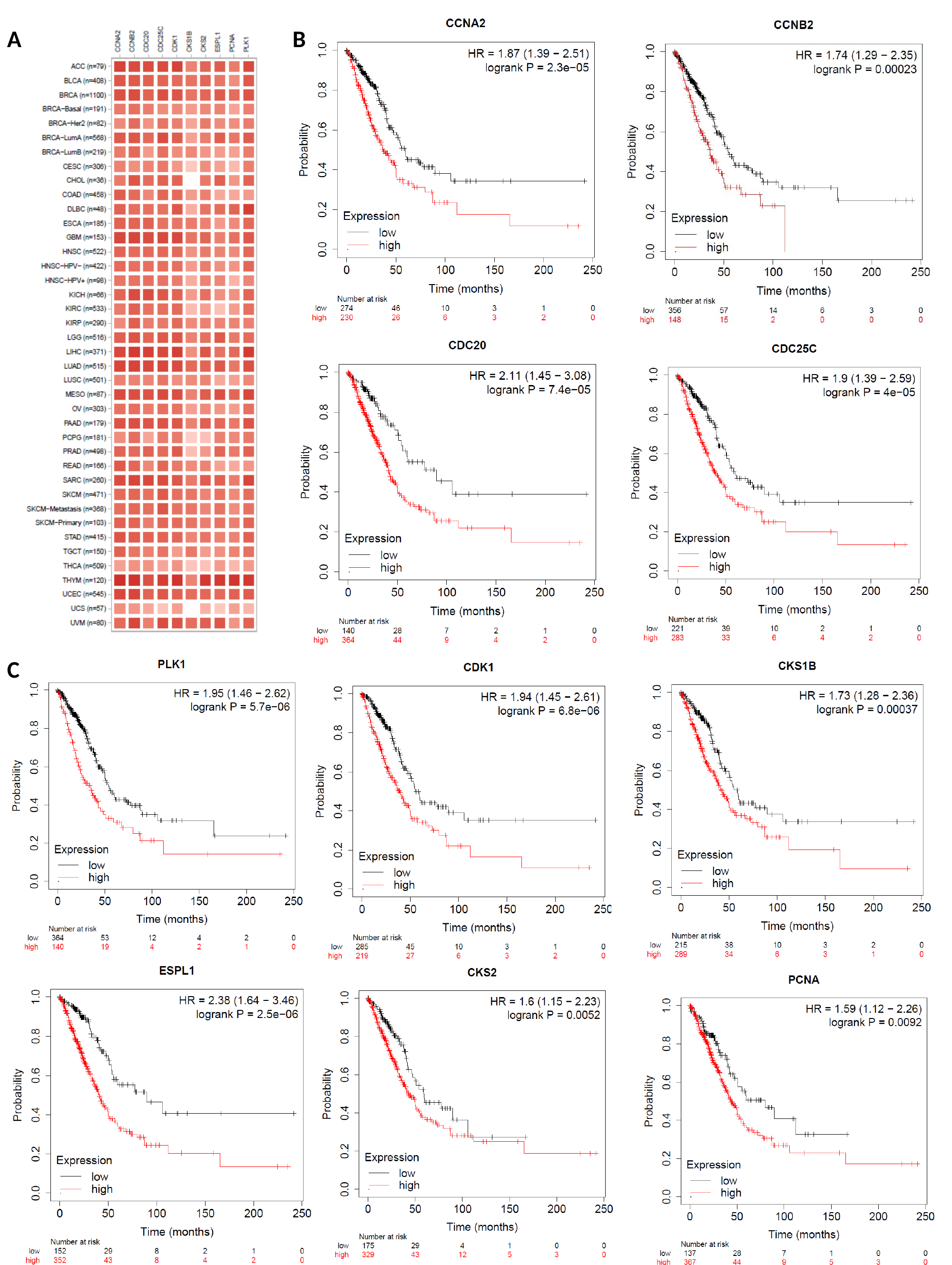

A
B
C

## Slide 4
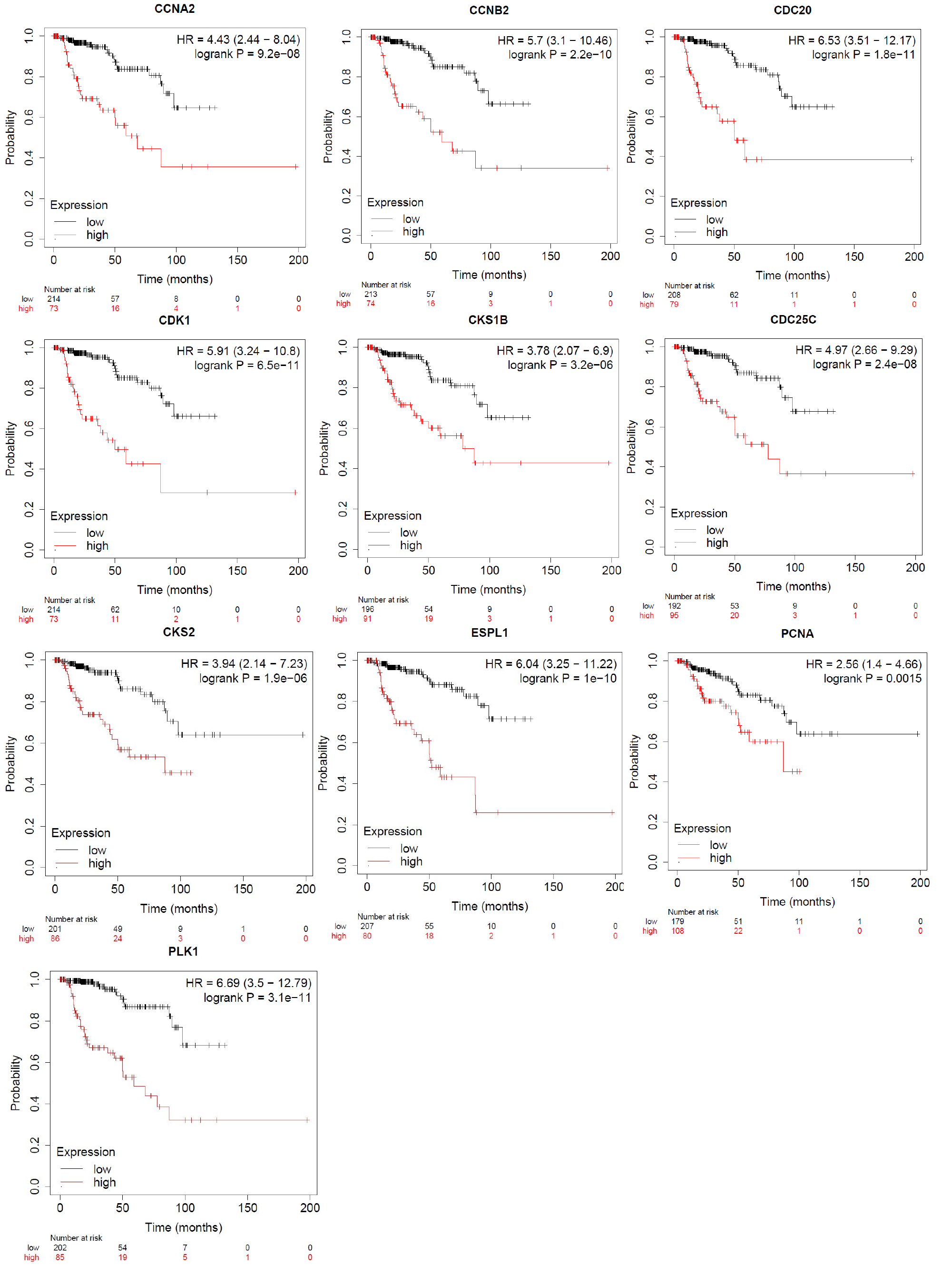

## Slide 5
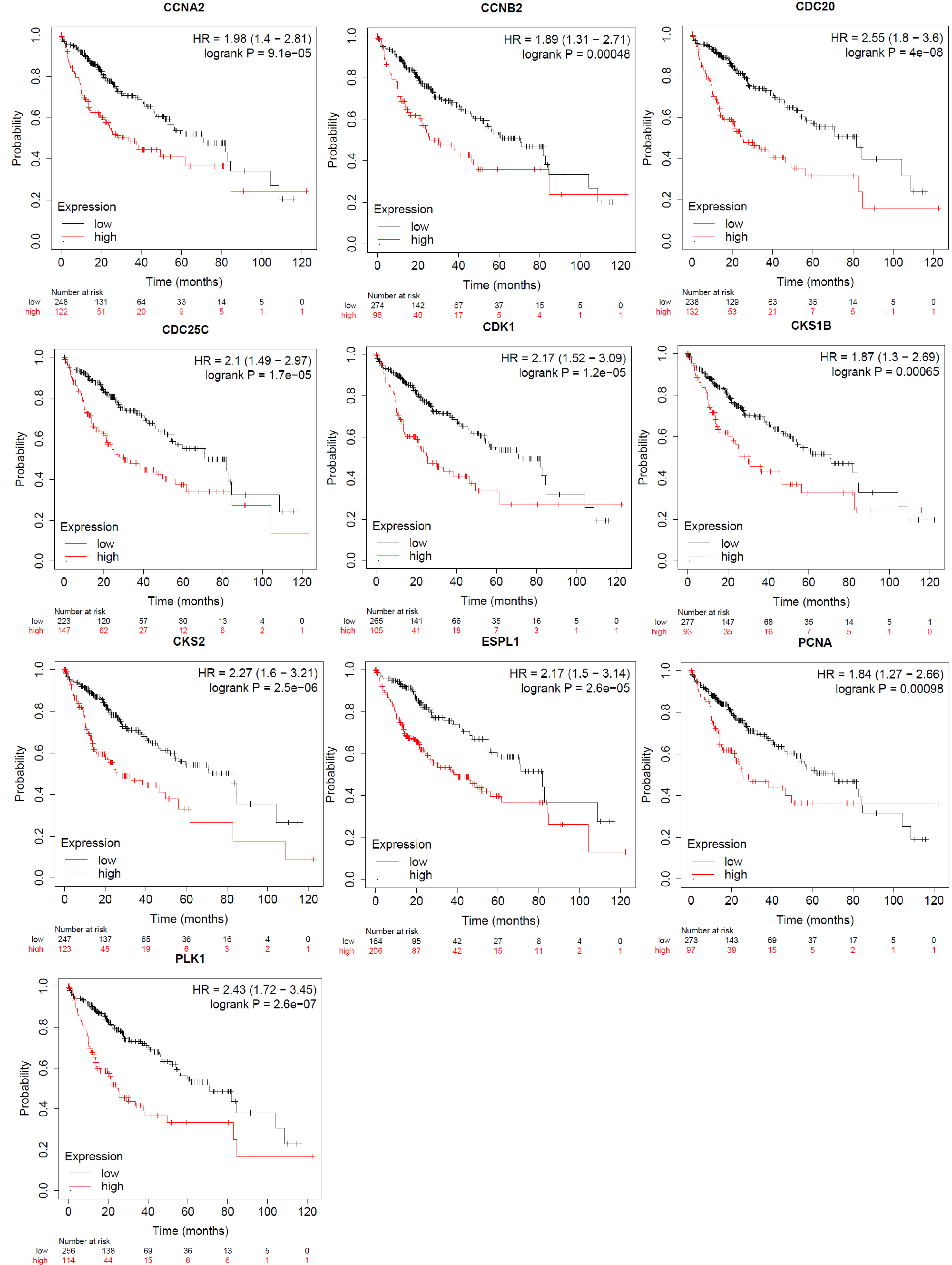

Supplement: Supplementary file 1 — Supplementary Figures. [file 41598_2023_42801_MOESM1_ESM.pptx]
